# Association between 1-year changes in urinary albumin-to-creatinine ratio and kidney disease progression in Japanese individuals with diabetes: a historical cohort study

**DOI:** 10.1007/s10157-023-02380-8

**Published:** 2023-08-22

**Authors:** Tetsuya Babazono, Ko Hanai, Yoichi Yokoyama, Kazuhisa Uchiyama

**Affiliations:** 1 Division of Diabetology and Metabolism, Department of Medicine, Tokyo Women’s Medical University School of Medicine, 8-1 Kawada-Cho, Shinjuku-Ku, Tokyo, 162-8666 Japan; 2 Clinical Development Department III, Daiichi Sankyo Co., Ltd., 1-2-58, Hiromachi, Shinagawa-Ku, Tokyo, 140-8710 Japan

**Keywords:** Chronic kidney disease, Type 2 diabetes, Urinary albumin-to-creatinine ratio, Estimated glomerular filtration rate

## Abstract

**Background:**

The National Kidney Foundation recently proposed a ≥ 30% decrease in urinary albumin–to–creatinine ratio (UACR) over 0.5–2 years as a surrogate endpoint for chronic kidney disease (CKD) progression in individuals with baseline UACR > 30 mg/g. This historical cohort study aimed to determine the applicability of a decrease in UACR, within as little as 1 year, as a surrogate endpoint for Japanese individuals with type 2 diabetes mellitus (T2D).

**Methods:**

A total of 5067 individuals with T2D were divided into three groups based on 1-year change in UACR: ≥ 30% decrease (UACR decreased group), < 30% decrease and < 30% increase (UACR unchanged group), or ≥ 30% increase (UACR increased group). The primary endpoint was a composite of a ≥ 30% decline in estimated glomerular filtration rate (eGFR) or the initiation of kidney replacement therapy, whichever occurred first.

**Results:**

At baseline, the proportions of individuals with normoalbuminuria, microalbuminuria, and eGFR ≥ 60 mL/min/1.73 m^2^ were 68.1%, 22.1%, and 75.5%, respectively. During a median follow-up of 6.8 years, 926 individuals (18.3%) reached the composite endpoint. Adjusted hazard ratios (vs. the UACR unchanged group) for the UACR decreased and increased groups were 0.758 (95% confidence interval [CI], 0.636–0.905; *P* = 0.002) and 1.304 (95% CI, 1.108–1.536; *P* = 0.001), respectively.

**Conclusions:**

These findings support the use of 1-year changes in UACR as a surrogate endpoint for the progression of CKD and the implementation of a ≥ 30% decrease in UACR as a positive efficacy endpoint in Japanese individuals with T2D and early-stage kidney disease.

## Introduction

Urinary albumin-to-creatinine ratio (UACR) and estimated glomerular filtration rate (eGFR) are essential for the diagnosis and staging of chronic kidney disease (CKD). We previously reported that a higher UACR at baseline was associated with a faster decline in subsequent eGFR in a historical cohort study of 5449 Japanese individuals with diabetes [1]. In a more recent cohort study of 8320 Japanese individuals with type 2 diabetes (T2D), we demonstrated worse kidney prognosis in individuals with low eGFR and normoalbuminuria than in those with normal eGFR, but better kidney prognosis than in those with albuminuria [2]. Considering other evidence from Japan [3], along with our two previous studies, UACR and eGFR at a specific time point play a pivotal role in predicting the risk of subsequent CKD progression in Japanese individuals. However, justifying the use of a clinical parameter as a surrogate endpoint for an interventional therapy necessitates robust evidence establishing a relationship between changes in the clinical parameter and the clinical outcome of interest [4]. On the basis of the meta-analyses of cohort studies and interventional studies [5, 6], the Scientific Workshop sponsored by the National Kidney Foundation (NKF), in collaboration with the US Food and Drug Administration and European Medicines Agency, proposed a ≥ 30% decrease in UACR within 0.5–2 years as a surrogate endpoint in clinical trials involving individuals with CKD, including early-stage CKD [7]. Implementing changes in UACR as the primary endpoint in clinical studies will facilitate the development of new therapies—particularly for early-stage CKD—by optimizing study durations and sample sizes.

However, the NKF meta-analyses included limited data from the Japanese population, necessitating further investigation to determine the applicability of these recommendations to the Japanese context. In our very recent study [8], we reported that a 3-year change in UACR served as a useful surrogate endpoint for kidney progression and mortality in Japanese individuals with T2D, including those with early-stage CKD. Nonetheless, the suitability of a shorter timeframe for changes in UACR as a surrogate endpoint is unknown. We thus conducted a further cohort study to test the validity of using changes in UACR during approximately 1 year as a surrogate endpoint for the progression of CKD in Japanese individuals with T2D.

## Methods

### Participants

This single-center historical cohort study was designed in adherence to the tenets of the Declaration of Helsinki. The Ethics Committee of Tokyo Women’s Medical University approved the protocol (approval no. 2020-0112), waiving the need for informed consent as this was a historical cohort study rather than a prospective interventional study. All participants were provided with the option to opt out through the website of Tokyo Women’s Medical University.

Initially, individuals with T2D who visited the Diabetes Center of Tokyo Women’s Medical University from August 1, 2003 to January 31, 2017, were identified from the hospital database and electronic medical charts. Among these, we included a total of 8,622 individuals who were aged ≥ 18 years, had not undergone kidney replacement therapy (KRT), and had measurements of body mass index, blood pressure, glycated hemoglobin (HbA1c), lipid parameters, serum creatinine, and UACR all obtained on the same day. If multiple measurements for variables were available within the study period, the earliest measurement was designated as the index date. We excluded individuals with the following conditions at the index date: pregnancy (*n* = 48), malignant diseases (*n* = 152), history of unilateral nephrectomy (*n* = 7), biopsy-proven diagnosis of non-diabetic kidney disease (*n* = 2), and acute kidney injury or post-renal failure (*n* = 2). We also excluded 3344 individuals who had no UACR measurement between 0.5 and 1.5 years after the index date (*n* = 3100), experienced a kidney outcome within 1 year after the index date (*n* = 181), had no serum creatinine measurements ≥ 1 year after the index date (*n* = 56), and had missing data on smoking status or height at the index date (*n* = 7). If multiple UACR measurements were available between 0.5 and 1.5 years after the index date, we selected the measurement closest to 1 year. Ultimately, 5067 individuals were eligible for inclusion in this study. Among them, 3497 individuals (69.0%) were also subjects in our recent cohort study examining the association between 3-year changes in UACR and the subsequent composite outcome of eGFR halving or initiation of KRT, and their index dates coincided with those in the present study [8].

### Study design

**Fig. 1:**
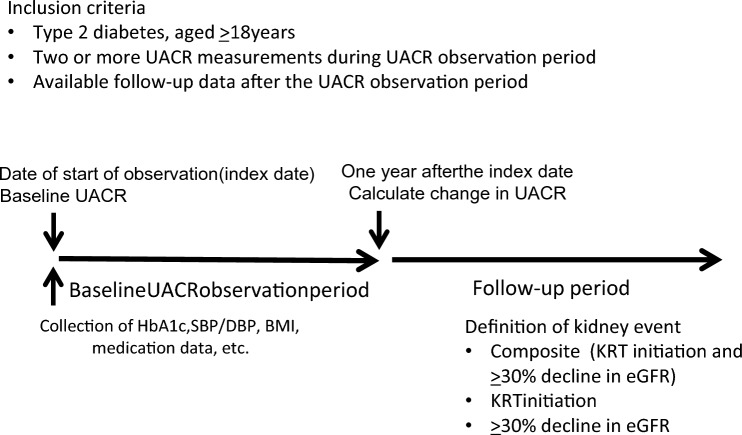
Study design. *BMI* body mass index, *DBP* diastolic blood pressure; *eGFR* estimated glomerular filtration rate; *HbA1c* glycated hemoglobin; *KRTI* kidney replacement therapy initiation; *SBP* systolic blood pressure; *UACR* urinary albumin–to–creatinine ratio

The study comprised two distinct follow-up periods: the baseline UACR observation period and subsequent follow-up period (Fig. 1). Based on the UACR change during the baseline period, individuals were classified into the following three UACR groups: a ≥ 30% decrease in UACR (the UACR decreased group), a < 30% decrease and < 30% increase in UACR (the UACR unchanged group), and a ≥ 30% increase in UACR (the UACR increased group). We selected the 30% threshold for UACR reduction based on previous studies demonstrating clinical benefit associated with a reduction in UACR of approximately 30% [7].

We defined a composite outcome of KRT initiation and a ≥ 30% decline in eGFR as the primary endpoint during the follow-up period. Analyses were also performed defining either KRT initiation or a ≥ 30% decline in eGFR as individual kidney events. The last observation day was January 31, 2018. Sensitivity analysis was performed by grouping individuals based on normoalbuminuria, microalbuminuria, and macroalbuminuria.

### Laboratory measurements

The first morning urine was used to measure UACR. eGFR was calculated using the following formula: eGFR (mL/min/1.73 m^2^) = 194 × age (years)^−0.287^ × serum creatinine level (mg/dL)^−1.094^ × 0.739 (if female), as proposed by the Japanese Society of Nephrology [9].

### Statistical analyses

Statistical analyses were performed using SAS Version 9.4 (SAS Institute Inc, NC, USA). Continuous data are expressed as arithmetic mean ± standard deviation or median and interquartile range (IQR), as appropriate. Categorical data are expressed as number (%). A two-tailed *P* value of < 0.05 was considered significant.

The cumulative incidence function and the multivariable Fine and Gray subdistribution hazard model were used, considering death as a competing event [10]. Change in UACR was also treated as a continuous variable, with the log_2_-transformed change in UACR modeled using the multivariable-adjusted restricted cubic spline function, where four knots were placed at the 5th, 35th, 65th, and 95th percentile levels. The following 14 variables were incorporated as covariates in the model: sex, age, presence of cardiovascular diseases, smoking history (current or ever), use of angiotensin-converting enzyme inhibitors and/or angiotensin II receptor blockers, body mass index, systolic and diastolic blood pressure, HbA1c, triglycerides (log-transformed), high-density lipoprotein cholesterol, low-density lipoprotein cholesterol, eGFR, and UACR (log-transformed) at the index date.

## Results

### Patient characteristics

The baseline characteristics of the 5,067 individuals at the index date are shown in Table 1. The mean age was 60 ± 12 years, and women accounted for 36.0% of the population. The mean HbA1c and eGFR were 7.6 ± 1.2% and 72.3 ± 21.3 mL/min/1.73 m^2^, respectively. The median UACR was 13.5 mg/g (IQR 6.7–47.5 mg/g). Among the participants, 75.5% had eGFR ≥ 60 mL/min/1.73 m^2^. The proportions of individuals with normoalbuminuria and microalbuminuria were 68.1% and 22.1%, respectively.

**Table 1:** Baseline patient characteristics (*N* = 5067)

Variables	All participants (*N* = 5067)	Change in UACR		
		Decreased (*n* = 1313)	Unchanged (*n* = 2061)	Increased (*n* = 1693)
Age, years (mean ± SD)	60 ± 12	60 ± 12	60 ± 12	61 ± 12
Women (*n*, %)	1824 (36.0)	545 (41.5)	692 (33.6)	587 (34.7)
Duration of diabetes, years [median (IQR)]	11 (5–18)	11 (5–19)	10 (5–18)	12 (5–19)
History of cardiovascular disease (*n*, %)	907 (17.9)	230 (17.5)	359 (17.4)	318 (18.8)
Smoker (current or ever) (*n*, %)	2681 (52.9)	666 (50.7)	1117 (54.2)	898 (53.0)
ACE inhibitor or ARB use (*n*, %)	2076 (41.0)	556 (42.4)	762 (37.0)	758 (44.8)
BMI, kg/m^2^ (mean ± SD)	24.9 ± 4.1	24.8 ± 4.1	24.9 ± 4.2	25.1 ± 4.1
Systolic blood pressure, mmHg (mean ± SD)	136 ± 20	137 ± 21	136 ± 19	136 ± 20
Diastolic blood pressure, mmHg (mean ± SD)	76 ± 12	77 ± 12	77 ± 11	76 ± 12
HbA1c, % (mean ± SD)	7.6 ± 1.2	7.7 ± 1.2	7.6 ± 1.2	7.6 ± 1.2
Triglycerides, mg/dL [median (IQR)]	122 (84–180)	122 (85–181)	121 (84–179)	122 (84–181)
HDL-C, mg/dL (mean ± SD)	54 ± 15	55 ± 15	54 ± 15	54 ± 15
LDL-C, mg/dL (mean ± SD)	114 ± 30	114 ± 31	115 ± 29	113 ± 30
eGFR, mL/min/1.73 m^2^ (mean ± SD)	72.3 ± 21.3	71.6 ± 21.6	73.6 ± 20.9	71.1 ± 21.6
≥ 60 mL/min/1.73 m^2^ (*n*, %)	3824 (75.5)	990 (75.4)	1597 (77.5)	1237 (73.0)
30–59 mL/min/1.73 m^2^ (*n*, %)	1076 (21.2)	268 (20.4)	409 (19.8)	399 (23.6)
< 30 mL/min/1.73 m^2^ (*n*, %)	167 (3.3)	55 (4.2)	55 (2.7)	57 (3.4)
UACR, mg/g [median (IQR)]	13.5 (6.7–47.5)	21.4 (10.0–82.4)	11.0 (6.3–29.5)	12.3 (5.6–47.1)
Normoalbuminuria (*n*, %)	3452 (68.1)	738 (56.2)	1549 (75.2)	1165 (68.8)
Microalbuminuria (*n*, %)	1117 (22.1)	413 (31.5)	334 (16.2)	370 (21.9)
Macroalbuminuria (*n*, %)	498 (9.8)	162 (12.3)	178 (8.6)	158 (9.3)

*ACE* angiotensin-converting enzyme, *ARB* angiotensin II receptor blocker, *BMI* body mass index, *eGFR* estimated glomerular filtration rate, *HbA1c* glycated hemoglobin, *HDL-C* high-density lipoprotein cholesterol, *IQR* interquartile range, *LDL-C* low-density lipoprotein cholesterol, *SD* standard deviation, *UACR* urinary albumin-to-creatinine ratio

During a median observation period of 1.02 years (IQR 0.88–1.17 years), 1313 individuals experienced a decrease in UACR (the UACR decreased group), 2061 individuals had unchanged UACR (the UACR unchanged group), and 1693 individuals had an increase in UACR (the UACR increased group). Other clinical characteristics and the main laboratory data for each UACR group are also summarized in Table 1.

### Summary of outcomes

**Fig. 2:**
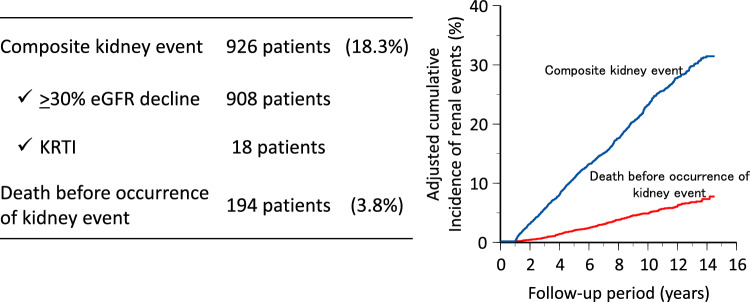
Outcome and cumulative incidence of kidney events (all individuals). *eGFR* estimated glomerular filtration rate; *KRTI* kidney replacement therapy initiation

**Fig. 3:**
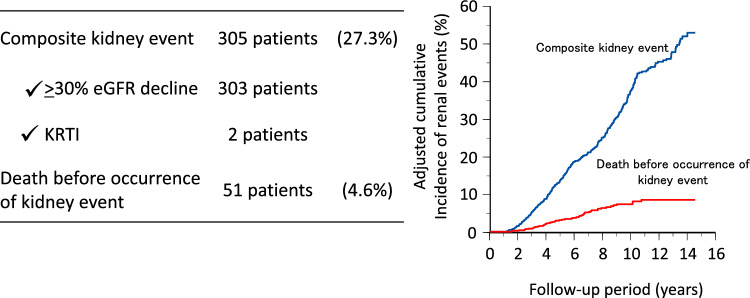
Outcome and cumulative incidence of kidney events (only individuals with microalbuminuria). *eGFR* estimated glomerular filtration rate; *KRTI* kidney replacement therapy initiation

During a median follow-up of 6.8 years (IQR 3.6–10.7 years), 926 individuals (18.3%) reached the composite kidney event endpoint, including 908 who experienced a ≥ 30% decline in eGFR and 18 who initiated KRT (Fig. 2). Among those who reached the ≥ 30% decline in eGFR, 172 individuals subsequently underwent KRT initiation. There were 194 deaths (3.8%) before the occurrence of a kidney event (a competing event). In the subgroup analyses limited to individuals with microalbuminuria, 305 individuals (27.3%) reached the composite kidney endpoint, including 303 individuals who reached a ≥ 30% decline in eGFR and two individuals who underwent kidney replacement therapy initiation (Fig. 3).

### Comparison of kidney outcomes among three groups classified by change in UACR

The adjusted hazard ratio (HR) for the composite kidney event in the UACR decreased group versus the UACR unchanged group was 0.758 (95% CI, 0.636–0.905; *P* = 0.002); that for the UACR increased group was 1.304 (95% CI, 1.108–1.536; *P* 0.001) (Table 2). The risks for each individual kidney event (≥ 30% decline in eGFR or KRT initiation) were consistently lower for the UACR decreased group and higher for the UACR increased group versus the unchanged group (Table 2).

**Table 2:** Association between change in UACR and kidney events: analyses for overall individuals and individuals with microalbuminuria

	Kidney event	Change in UACR	Individuals (*n*)	Follow-up duration (year) × individuals (*n*)	Kidney events (*n*)	Events/1000 individuals × year (*n*)	Adjusted HR	95% CI	*P* value
Overall individuals (*n* = 5067)	Composite	Decreased	1313	9333	243	26.0	0.758	0.636–0.905	0.002
		Unchanged	2061	15,452	341	22.1	Ref	–	–
		Increased	1693	11,789	342	29.0	1.304	1.108–1.536	0.001
	≥ 30% eGFR decrease	Decreased	1313	9333	236	25.3	0.746	0.625–0.890	0.001
		Unchanged	2061	15,452	338	21.9	Ref	–	–
		Increased	1693	11,789	334	28.3	1.293	1.097–1.524	0.002
	KRTI	Decreased	1313	10,159	48	4.7	0.622	0.410–0.945	0.026
		Unchanged	2061	16,573	68	4.1	Ref	–	–
		Increased	1693	13,010	74	5.7	1.749	1.210–2.526	0.003
Individuals with microalbuminuria (30 ≤ UACR < 300 mg/g) (*n* = 1117)	Composite	Decreased	413	2975	91	30.6	0.736	0.557–0.973	0.032
		Unchanged	334	2400	102	42.5	Ref	–	–
		Increased	370	2241	112	50.0	1.283	0.983–1.676	0.067
	≥ 30% eGFR decrease	Decreased	413	2975	90	30.2	0.725	0.548–0.960	0.025
		Unchanged	334	2400	102	42.5	Ref	–	–
		Increased	370	2241	111	49.5	1.273	0.975–1.663	0.076
	KRTI	Decreased	413	3330	5	1.5	0.491	0.157–1.542	0.223
		Unchanged	334	2741	7	2.6	Ref	–	–
		Increased	370	2696	21	7.8	2.478	1.021–6.019	0.045

*CI* confidence interval; *eGFR* estimated glomerular filtration rate; *HR* hazard ratio; *Ref.* reference; *KRTI* kidney replacement therapy initiation; *UACR* urinary albumin-to-creatinine ratio

Similar trends to those of the overall analysis were observed in the sensitivity analysis of 1117 individuals with microalbuminuria.

### Analyses treating change in UACR as a continuous variable

**Fig. 4:**
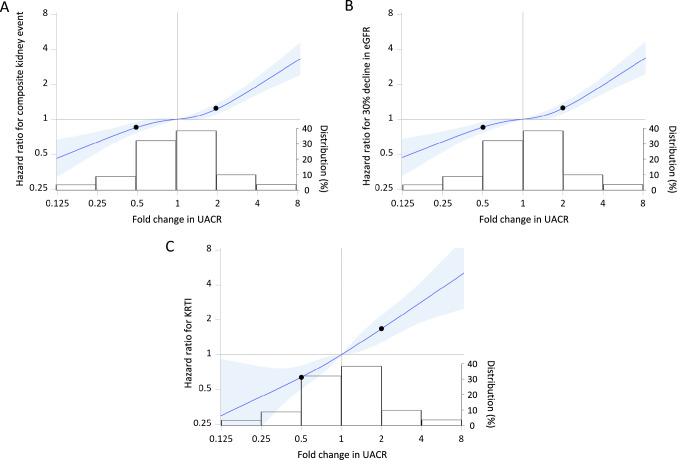
Multivariable-adjusted restricted cubic spline curves of the association between kidney events and change in UACR, with histograms of change in UACR. **A** Composite kidney event, **B** ≥ 30% eGFR decline, **C** KRTI. In Panel **A**, the left and right black circles show HRs at halving (0.855 [95% CI, 0.767–0.952]) and doubling (1.239 [95% CI, 1.107–1.387]) of UACR, respectively. In Panel **B**, the left and right black circles show HRs at halving (0.855 [95% CI, 0.768–0.953]) and doubling (1.246 [95% CI, 1.113–1.396]) of UACR, respectively. In Panel **C**, the left and right black circles show HRs at halving (0.640 [95% CI, 0.504–0.811]) and doubling (1.673 [1.274–2.195]) of UACR, respectively. *CI* confidence interval, *eGFR* estimated glomerular filtration rate, *HR* hazard ratio, *KRTI* kidney replacement therapy initiation, *UACR* urinary albumin–to–creatinine ratio

The association of UACR fold change with the composite kidney event outcome was generally linear (Fig. 4A). On the spline curve, the HRs for the composite kidney event outcome at halving and doubling of UACR were 0.855 (95% CI, 0.767–0.952) and 1.239 (95% CI, 1.107–1.387), respectively (Fig. 4A). A similar result was observed in the separate analyses of the ≥ 30% decline in eGFR and KRT initiation (Fig. 4B, C).

### Comparison of kidney outcomes among three groups classified by baseline UACR

**Fig. 5:**
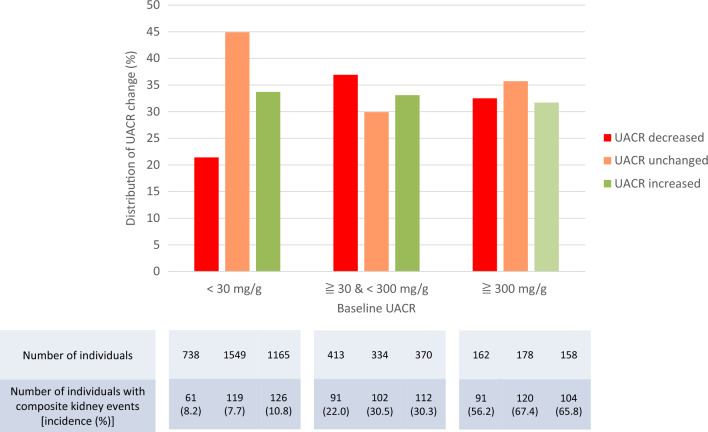
Distribution of UACR change and number of occurrences of composite kidney events by albuminuria stage at baseline. UACR decreased: ≥ 30% decrease in UACR; UACR unchanged: < 30% decrease and < 30% increase in UACR; UACR increased: ≥ 30% increase in UACR. *UACR* urinary albumin-to-creatinine ratio

When individuals were classified according to baseline albuminuria, the incidence of composite kidney events in the UACR decreased individuals was lower than that in the UACR increased individuals in all the three groups classified (Fig. 5).

## Discussion

The results of the current cohort study not only confirmed our recently published data but also provide additional findings demonstrating a positive correlation between a decrease in UACR and kidney events—even over a shorter timeframe of approximately 1 year. The findings suggest that changes in UACR over 1 year can be used as a reliable surrogate marker for evaluating the efficacy of interventions in this population. Consistent results were observed when analyzing a subgroup of participants exhibiting microalbuminuria, indicating the robustness of the trend.

Traditionally, initiation of KRT and doubling of serum creatinine levels have been established as efficacy endpoints in clinical studies involving individuals with CKD. However, these endpoints generally require long-term observation and large sample sizes, limiting eligibility to individuals with late-stage CKD. Recently, the Scientific Workshop sponsored by the NKF proposed a ≥ 30% or a ≥ 40% decline in eGFR as an efficacy endpoint in addition to the conventional kidney events [11]. A similar proposal has also been made by the Japanese Society of Nephrology [12]. These proposals may facilitate the development of new drugs primarily for individuals with overt albuminuria. However, the development of new drugs for individuals with microalbuminuria still poses significant challenges.

The initiation of interventional therapy for individuals with early-stage CKD is believed to be more efficient in ensuring the prevention or delay of the transition to end-stage kidney disease. The establishment of surrogate endpoints specifically targeting this population is thus urgently required. To address this, the workshop sponsored by the NKF performed meta-analyses using cohorts and interventional studies, aiming to identify alternative efficacy endpoints applicable to clinical studies involving individuals with early-stage CKD [5, 6]. Their analyses demonstrated that a change in UACR over a period of 0.5–2 years was significantly associated with the occurrence of subsequent kidney events, both in individuals with macroalbuminuria and in those with microalbuminuria. On the basis of these results, the workshop proposed that a ≥ 30% decrease in UACR could serve as a surrogate endpoint, indicating a positive efficacy signal, across various cohorts [7]. Following the proposal, several placebo-controlled double-blind clinical studies evaluating medications for CKD in diabetes, such as mineralocorticoid receptor antagonists (MRAs) and sodium glucose co-transporter 2 (SGLT2) inhibitors demonstrated correlations between changes in UACR and the incidence of kidney events [13–15]. These findings further supported the proposal of the NKF-sponsored workshop [7].

In our recent study [8], we demonstrated that a 3-year change in UACR can serve as a surrogate endpoint for Japanese individuals with T2D, including those with early-stage CKD. However, using a baseline observation that requires long-term observation may not contribute significantly to the efficient development of new interventional therapies. To reduce drug development costs and minimize participant burden, it is desirable to explore shorter durations of baseline observation. To address this, we conducted the current study to evaluate the applicability of the recommendations of a ≥ 30% reduction in UACR over a shorter period to Japanese individuals with T2D. Our database consists of individuals with T2D, encompassing a considerable number of cases with both normoalbuminuria and microalbuminuria, making it an ideal database for investing individuals with early-stage CKD in the context of diabetes. Our findings clearly demonstrated that a change in UACR—even over a shorter period of approximately 1 year—was significantly associated with the occurrence of the composite kidney event. These associations remained consistent when analyzing KRT initiation and ≥ 30% decline in eGFR as separate outcomes. Moreover, similar trends were observed in the sensitivity analyses conducted specifically in individuals with microalbuminuria, although statistical significance for individual event HRs was not consistently reached because of small sample sizes in some analyses. While our current study and previous study [8] used a common pool of patients extracted from the same database, the current study, with its distinct research objectives and different criteria for defining eGFR decline, sheds light on the applicability of a shorter timeframe for changes in UACR as a surrogate endpoint, potentially facilitating the future design and conduct of interventional studies.

Further analyses from the NKF workshop demonstrated that a reduction in eGFR slope by 0.5–1.0 mL/min/1.73 m^2^/year over 2–3 years was also associated with a reduced risk of subsequent kidney events. This suggests that the reduction in eGFR slope could serve as a positive efficacy endpoint [16, 17]. It is worth noting that the number of individuals with diabetes who experience kidney function decline without following the typical progression from normoalbuminuria and microalbuminuria to macroalbuminuria is increasing [18–20]. Considering this, using eGFR slope as an efficacy endpoint for such individuals seems reasonable. However, it is important to consider that certain medications, including angiotensin II receptor blockers, SGLT2 inhibitors, and MRAs, can cause acute declines in eGFR, which may affect the assessment of efficacy when using eGFR slope as an endpoint [13–15, 21–24]. For example, in a clinical study of finerenone, a greater decrease in eGFR was observed in the finerenone group than in the placebo group during the first 4 months of treatment [13]. Subsequently, the decline in eGFR in the finerenone group became more gradual than that in the placebo group by the end of the treatment period. However, when considering the entire treatment period, including the first 4 months, the between-group difference in eGFR slope was estimated to be < 0.5 mL/min/1.73 m^2^/year, which is less than the minimum threshold of eGFR slope recommended by the NKF workshop. Given these observations from the finerenone study, caution is required when using eGFR slope as an efficacy endpoint if the early decline in eGFR caused by medications is included in the assessment.

This study has several limitations that should be acknowledged. First, the findings may have limited generalizability to all Japanese individuals with T2D, as the study was conducted at a single center, introducing selection bias. Therefore, caution should be exercised when extrapolating the results to other populations. Second, the lack of information on medication use, including SGLT2 inhibitors and MRAs during both the baseline UACR observation period and subsequent follow-up period, is a notable limitation. These medications could potentially have had an impact on the observed results. Third, UACR values at the index date and end of the observation period were determined based on a single measurement, which may have led to improper categorization because of the day-to-day variability in albumin excretion [7, 25]. While multiple measurements of UACR would have provided a more accurate assessment of albuminuria, such repeated measurements were not obtained. To mitigate this limitation, the timing of urine collection was restricted to the first morning urine, minimizing the potential influence of exercise-induced variations and diurnal fluctuations on albumin excretion. Finally, it is important to note that there may be additional covariates that were not included in the multivariate analysis, which could potentially influence the outcomes.

## Conclusions

This historical cohort study, conducted in Japanese individuals with T2D, provides evidence that changes in UACR over a period of approximately 1 year were significantly associated with a long-term kidney prognosis, i.e., a composite of ≥ 30% decline in eGFR and KRT initiation, independently of clinical parameters including baseline UACR and other clinical characteristics. These findings support the use of the change in UACR over a 1-year period as a surrogate endpoint for assessing kidney events in clinical studies involving individuals with early-stage CKD and diabetes. Furthermore, the results support the recommendation of a ≥ 30% decrease in UACR as a positive efficacy endpoint for evaluating interventions in Japanese individuals with T2D and early-stage CKD.
